# Midwives’ Perspectives on Family Planning With Pregnant and Postpartum Women in the Philippines’ Mindanao Region: A Qualitative Study

**DOI:** 10.7759/cureus.26473

**Published:** 2022-06-30

**Authors:** Alexandra Norton, Nicole A Shilkofski

**Affiliations:** 1 Pediatrics, Johns Hopkins University School of Medicine, Baltimore, USA

**Keywords:** qualitative research, midwifery, philippines, contraception, family planning

## Abstract

Background

This study sought to identify factors significantly impacting access to and utilization of modern contraceptive methods among Filipinas from the perspective of midwives who are caring for women during pregnancy and the postpartum period.

Methods

In-depth, semi-structured interviews were conducted with 10 midwives working at three birthing clinics within the Mindanao region of the Philippines. Data were coded and analyzed for major themes using a grounded theory approach.

Results

Logistics of obtaining the desired contraceptive method, superstitions associated with family planning (FP) methods, opinions of community leaders and partners, and education regarding fertility and birth spacing benefits are significant factors impacting Filipina family planning use. Religion and midwives’ preferences are not significant factors.

Conclusions

Cultural and social factors play a large role in family planning decisions. There is a need for enhanced education, beginning in schools, regarding the fertility cycle, birth spacing benefits, and the importance of individual family planning. Filipino public health infrastructure operating consistently is a key factor for women to reliably access contraception.

## Introduction

While the global maternal mortality rate decreased by 38% from 2000 to 2017, much progress is still needed [1]. The need for progress can be seen clearly in the Philippines, with a 2017 maternal mortality rate of 121 maternal deaths per 100,000 live births, nearly twice the regional average [2]. Within the Philippines, the Mindanao region’s maternal mortality rate is higher than the national average and increasing (Molleno LJ, personal communication, July 2016).

The Mindanao Department of Health has robust efforts to address maternal mortality, which includes a goal of increasing the contraceptive prevalence rate from 54% to 65%, recognizing that the use of modern contraception decreases maternal mortality (Molleno LJ, personal communication, July 2016). Contraception access in low-income countries has been shown to decrease high-risk births from high parity, shorter birth intervals, and advanced maternal age [3]. The World Health Organization identifies the following as modern contraceptive methods: combined oral contraceptives, progestin-only pills, implants, injectables, patches, rings, intrauterine device (IUD), male or female condoms, male or female sterilization, lactational amenorrhea method, emergency contraception, standard days, basal body temperature, two day, and sympto-thermal methods. Traditional methods include the calendar/rhythm method and withdrawal [4]. There is evidence that women who receive family planning (FP) information at the time of delivery are more likely to subsequently utilize modern contraception [5,6]. Aiming to broaden the base of providers skilled in administering all modern contraceptive methods, programs for Mindanao-based midwives have included FP competency-based and contraceptive technology training (Molleno LJ, personal communication, July 2016). These efforts exist within a complex cultural landscape. The 2012 Responsible Parenthood and Reproductive Health Act aimed to increase contraceptive access; however, conservative sociocultural norms and historical governmental and religious opposition to modern contraception have remained obstacles [7].

The use of modern contraception among Filipinas has been slow to increase [8]. Contraceptive supply stock-outs and other inventory factors are cited as availability challenges [9]. Other barriers include finances, difficulty accessing services, cultural factors, and gender roles [8,10]. A lack of sexual education, specifically limited understanding of the fertility cycle, has been noted [8]. Religious beliefs, lack of desire to use contraception, and lack of knowledge of contraceptive methods have not been identified as barriers [8,11].

Several qualitative studies have queried Filipinas about contraceptive access and utilization [12-14]. A separate body of literature cites the integral role Filipina midwives play in women’s health service provision, as the primary providers of health services in neighborhood-based clinics [10,11]. However, despite the centrality of midwives in both counseling regarding and provision of contraception, there has not been comparable literature presenting qualitative data from a midwife’s perspective. As efforts are made to impact the use of modern contraception in the Philippines, the insight of the service providers closest to the issue is a critical component that to date is missing in the literature. The objective of this study was to assess factors impacting access to and utilization of modern contraception among women in the Mindanao region, from the perspective of midwives who counsel women regarding FP during pregnancy and in the postpartum period.

## Materials and methods

Participants and study setting

Midwives and nurse-midwives employed at the Mercy Maternity Clinic in Davao City, Mindanao, and the Mati Balay Paanakan in Mati City, Mindanao, participated in this study (n=10). The three birthing clinics at which participants were employed were in both urban and rural areas of Mindanao, located in the southern portion of the island chain in the Philippines. The urban clinic was a private charity clinic. The two rural clinics were public birthing clinics and part of the national Barangay Health Station (BHS) network, which provides neighborhood-based primary care services throughout the country.

Utilizing purposive sampling, the participants were recruited in person at their sites of work by a single external investigator. The participants were recruited during work shifts, as patient volume permitted, and all possible participants who were approached consented to be interviewed. Each participant provided verbal consent to be interviewed and audio-recorded. The participants were informed of their right to end the interview at any time or to withdraw their participation. All participants were from the Mindanao region and received their midwifery training in the Philippines. The interviews lasted between 13 and 71 minutes. One participant expressed discomfort during the interview, and the interview was promptly ended. This participant consented following the interview for the recording to be included in the study, so all interviews were included in the data analysis.

Procedure

This study was approved by the Institutional Review Board of the Johns Hopkins University School of Medicine (JHUSOM). The qualitative study was conducted using an ethnographic and grounded theory approach. In-depth, semi-structured interviews were conducted in English by a single investigator as either one-on-one or one-on-two conversations in June and July of 2018. Possible areas of inquiry were identified through a literature review, and one week of field observation further informed the development of an interview guide. This guide served as the core question set for each interview. All interviews were audiotaped for purposes of subsequent transcription and coding for thematic analysis, with the audio recording transferred to password-protected storage and deleted from the recording device following the interview. Interviews were conducted until thematic saturation was reached. Observational data were collected from interactions between the midwives and their patients for purposes of triangulation.

Funding support was received from the JHUSOM’s Dean’s Summer Research Fund for travel-related expenses for the primary investigator. The funder played no role in either conducting research or writing this manuscript.

Data analysis

All interviews were transcribed by an external transcription service, TranscribeMe, and reviewed for accuracy by the primary investigator. The interview data were coded and analyzed for major themes, using the Dedoose qualitative analysis platform. An open coding process was followed, with the utilization of initial open coding categories. The subcategories from each initial category were connected in the process of axial coding, through which the study’s major themes and subthemes emerged. The original interview recordings were available throughout the analysis process.

Following the identification of major themes and subthemes, “thick description” was applied for each theme to identify representative quotations from the original data. This thick description allows for the voice of the midwives and nurse-midwives to be conveyed, offering valuable context.

Data for the purposes of triangulation were collected throughout the primary investigator’s time in the participating clinics, with observed encounters between midwives and patients from prenatal through labor and postpartum visits. These informal observations were consistent with the study’s ethnographic approach and served to corroborate and validate the interview findings.

## Results

Ten midwives and nurse-midwives were interviewed for this study. Information was solicited during these interviews to characterize both midwife and patient preferences regarding FP methods. The midwives were asked to both reflect on their own FP recommendations and the methods their patients most often ask about and reference using; the findings are summarized in Table 1. The midwives were also asked about the timing of FP counseling conversations with their patients, and 70% said that FP counseling occurred during prenatal visits, with 60% providing FP counseling during the delivery visit and 90% during the postpartum visit.

**Table 1:** Midwives’ Preference and Midwife-Identified Patient Interest for Specific Family Planning Methods

Method	Midwives’ preference (%) (n=10)	Midwife-identified patient interest (%) (n=10)
All methods	40%	-
Condoms	0%	20%
Implant	20%	30%
Injectable (Depo)	30%	60%
IUD	50%	40%
Oral contraceptives	0%	90%
Traditional method	0%	70%
Tubal ligation	10%	50%

Four primary themes emerged from interview coding as having a significant impact on access to and utilization of modern contraception. Additionally, there are notable factors that emerged as not significantly affecting patterns of modern contraception use. These themes and notable subthemes are explored below and in Table 2, Table 3, and Table 4.

**Table 2:** Themes for Factors Impacting Family Planning (Representative Quotations From Interviews)

Logistics	
Referral logistics	Oh, we don’t have family planning here, but we can refer you to the health center, and if you really want, you can go to your nearest health center.
Resource availability	Usually, the patient will tell me, “Ma’am, the health center is out of stock.” It depends on the availability and where they are getting it. Not everything they can get. Maybe they have only pills, and they don’t have other methods.
Appropriate training	Before we have a trained midwife here, used to insert an IUD, Implanon, but since they’re gone, we don’t have any family planning except for the oral contraceptive.
Finances	Sometimes, they worry that it is expensive, but then, when they learn that it is free, they become interested in family planning. As long as you have PhilHealth, it’s free.
Superstitions and fear	
Superstitions	In all types of family planning, there are some kinds of superstitions and rumors. That’s why lots of patients are not interested in family planning.
Fear	They want to (use family planning), but they’re scared of these things because that’s what they heard.
Neighbors and banas	
Neighbor opinion	They’re like, “My neighbor is very confident in telling me about this.” Every time, teaching is like, “Your neighbor, maybe you can bring them here. I will educate them because they’re a very big influence to many.”
Bana opinion	Normally, they cannot decide for themselves. There will be a consultation with a bana. Our practice here in the Philippines is that you have to talk to your partner about it.
Education gaps	
General sex and FP education	They don’t have enough knowledge of family planning. We need to counsel them more.
Benefits of and maternal health considerations for family planning	We inform them of the importance and the benefits of having family planning. We talk to them (about) the benefits for the family, community, mother, children, and especially, the dad. They need time to recover. We also explain what might happen if, after a year, they will get pregnant, and it will cause also bleeding. (I) explain to them that at least you can give time for your partner and your baby because if you get pregnant again next year and next year and next year, then how will you take care of yourself or your baby?
Fertility timing	They think that they will not get pregnant at six weeks (postpartum) because, usually, they thought that after six weeks, that’s the time that the menstruation comes.
Correct utilization of family planning method	Some women here, if they are using pills, maybe, don’t have any counseling. That’s why they missed. They became pregnant. “I’m using that pills, but I got pregnant.” Then we asked, “Why did you get pregnant?” It’s because they forgot to take it, because they did not undergo counseling. You can always use pills, but make sure that you won’t forget them, and you need to take them at the same time.

Logistics of obtaining desired family planning method

The midwives reported that the requisite logistics for their patients to obtain their desired method often prove challenging and prohibitive. Patients are not typically able to access contraception at the time of delivery, and midwives described referring patients to their BHS following delivery. Patients visit their BHS in the weeks following delivery to access immunizations for their baby, but midwives noted that patients “go for the baby’s immunization, but then, sometimes, they forget the FP.”

When patients are able to visit their BHS for FP services, midwives reported variability in the methods available. When asked if patients could go to a different BHS if needed for their preferred method, the response was, “Usually, they’re not giving if you’re not living in that barangay.” The midwives described that patients then would either need to wait or, if medically and financially possible, purchase in a drug store.

Midwives similarly reported variability in the training of BHS midwives for inserting IUDs and implants. Some interviewees reported that “most of the midwives in the community now is [sic] already trained,” while another said of herself and a fellow midwife, “We are only two that [sic] was trained in the implant. Some of my colleagues call me to go to their community to offer FP.”

The cost was described by the midwives as more of a perceived than an actual barrier. They referenced changes introduced by the 2012 bill in describing past affordability challenges and that some patients still assume they won’t be able to afford it. Some midwives also cited factors other than the cost of the method itself, such as professional fees or fees for transportation to the BHS.

Superstitions and fear regarding modern contraceptive methods

The midwives reported that there are superstitions associated with most modern contraceptive methods that contribute to the fear of method utilization. The midwives all used “superstition” as the descriptor for FP-related misconceptions, and that language is retained in this description of the results. Midwives cited specific superstitions for each of the modern methods, which are summarized in Table 3. Some also said that patients prefer traditional FP “because of the misconceptions” around modern contraceptive methods.

**Table 3:** Superstitions Held by Patients Regarding Family Planning Methods

Methods	Superstitions
IUD	Causes pain for the partner, unable to lift heavy things or do household chores, causes heavy bleeding
OCP	Causes cancer and tumors
Tubal ligation	Major surgery, unable to lift heavy things, causes increased sexual arousal and interest in other partners
General	Blood being trapped in the uterus if menstruation is altered by the FP method

Many midwives referenced fear both stemming from and independent of superstitious beliefs. Fear was most often mentioned in the context of procedural contraceptive options, saying “most of the patient has [sic] a fear of using IUD,” and regarding tubal ligation, “they’re really scared of the operation.” These fears made contraceptive pills an attractive option in comparison; “it’s more safe [sic], and it’s easy. Maybe, some of them are afraid also, like the procedure, and the pills are [sic] just drink.”

The midwives described a range of approaches to and success in addressing these misconceptions and fears. One midwife described, “It’s very hard sometimes to educate patients, especially when they have this already instilled in their minds.” Another said, “Most of the patients, really, they know better, and then, they just worry that it’s false.” We used to tell them “Oh, where is the evidence? No study proved that one. Most of them are going to believe that.” Several midwives described more success with addressing the fear that comes from the procedures themselves rather than superstitions, with one saying, “You just have to explain to them really well until they understood [sic], every single information that they need to know so that the fear will be minimized.”

Neighbor and bana (partner) opinions

Opinions held by “neighbors,” or influential community members, along with that of the “bana” or partner, are often cited as a significant factor in an individual’s FP decision. Several midwives spoke both about the influence of neighbor opinions and the need for broader community education to address the effects of misinformation that is easily spread. One midwife stated, “Here in the Philippines, we used to listen to our neighbors, what is the talk of our neighbors about the FP. So, I used to educate them that you have to go to me. Do not go to your neighbor.” Another put it, “Neighbors, mothers-in-law, I want to talk to all of them.”

Similarly, the bana is consistently named as having a vital role in FP decisions. Midwives described banas expressing strong opinions regarding certain FP methods and specifically the IUD, because of superstitions regarding pain during intercourse. Overall, the bana is described as being “quiet” in FP conversations at the birthing clinics, with some “who will ask what are [sic] the nice, good FP to use.” One midwife described that it is “usually the woman who plans to have the FP, takes much more initiative than the bana” but that a collective decision is made, and generally, the couple will “go together” to the BHS to access FP. Another midwife explained that she is “very strict to counsel both the couple so that they can have the same overlook about the situation.”

Education gaps related to family planning

The midwives raised numerous relevant education gaps that they see having an impact on their patients’ FP decisions and success. First, they identified gaps in, generally, sex and family planning education. They described that sex education is provided in schools, but students don’t “apply” this knowledge, and they often help patients to learn about FP options.

Honing in on a more specific aspect of educating about FP, the midwives identified conveying the benefits of FP to be one of their main goals of counseling. There is also a particular focus on the maternal health aspect of FP, and one midwife described this educational point, saying, “Usually, here in the Philippines, we have at least two years spacing so that they can rest and make time for family, but also, the uterus will rest [sic] during these two years.”

Many midwives described a gap in patient knowledge regarding fertility timing and return of fertility following delivery. Most midwives referenced conversations where patients had assumed that they could not get pregnant again before menstruation returned. Midwives described patients forgetting or deciding to wait until later to go to the BHS for FP, and they cited education around return of fertility as a critical message starting during prenatal counseling.

Midwives also frequently referenced incorrect use of a particular FP method as the reason for an unplanned pregnancy. The most commonly cited was inconsistent adherence to oral contraception. This was mentioned as a critical education point as well, reinforcing the need to take pills consistently.

Factors that do not impact family planning

Several factors consistently arose in the interviews as not significantly impacting FP (Table 4). Religion was discussed in all interviews, and the midwives consistently reported that Christian or Catholic religion was not a barrier to patients using modern contraception. The midwives reported a wider range of perspectives regarding Muslim patients, some citing a lower rate of FP use among Muslim patients but that most patients would say “that it is up to them.”

Overall, the midwives expressed a desire to counsel patients on the full spectrum of FP options, saying, “I throw everything out there.” While several explained their way of telling patients about more and less effective methods; none expressed unwillingness to counsel on any particular method.

**Table 4:** Themes for Factors Not Impacting Family Planning (Representative Quotations From Interviews)

Religion	
Christianity	The Christians are allowed. They are more open.
Islam	Before, their imams prohibited them to use any method. It’s their practice with Islam (not to use family planning), but what I say, the new generation of Muslims now, they’re open-minded now.
Midwives’ preference	I am trying to give them an insight about other family planning methods … we must introduce (them) to the whole.

Other factors

A number of additional factors impacting FP came up during the interviews but were not consistently cited as playing a significant role in patients’ FP considerations. These included the following: the patient and the bana’s education level, limited time for FP counseling during patient encounters, side effects of FP methods, and utilization of traditional birth attendants. At one of the clinics, the Badjao, a minority ethnic community, were described as having FP practices that differed from the majority of the patients. As this was a small minority of patients at one clinic, these comments were omitted from the broader thematic review.

## Discussion

Main findings and interpretation

This study identified four primary themes impacting access to and utilization of modern contraception. The logistics of obtaining FP are often prohibitive as patients must visit their BHS following delivery and available methods vary, due to inventory fluctuations and differences in BHS provider training. Superstitions associated with modern contraceptive methods contribute to fear of method utilization. Opinions held by “neighbors” and “banas” are significant factors in FP decisions, emphasizing the weight of communal opinions. FP education is a priority for midwives and is seen as a critical knowledge gap for many patients. Patients’ religious beliefs and midwives’ method preferences were not reflected as significantly impacting FP decisions.

Challenges of increasing the use of and addressing the unmet need for modern contraception in the Philippines have been well documented in health statistics and studied in the literature from numerous perspectives [7,8,15,16]. According to the 2017 national data, 40% of currently married women and 17% of sexually active unmarried women are using a modern contraceptive method. Modern contraceptive use increased just 2% from 2013 to 2017 [15]. Framing the need differently, 54% of pregnancies in the Philippines are unplanned [16]. Furthermore, a 2015 study found that 50% of postpartum Filipina women have short interpregnancy intervals, finding only 34% of Filipinas used FP two years postpartum [17].

Existing literature has identified numerous contributing factors to these trends, many of which this study reinforced from the midwives’ perspectives. Many studies have highlighted gaps in modern contraception availability. A 2019 study found a consistent supply of short-acting contraceptive methods in surveyed BHS locations but sparse long-acting contraceptive availability [18]. Another study cited a need for improved local data, as current tracking underestimates contraceptive supply needs in specific communities [9]. This study echoed these findings, as midwives reported their patients’ challenges in accessing their desired contraceptive method at their assigned BHS.

Supply availability must be paralleled by the availability of providers equipped to counsel in contraceptive use and trained in the application of certain methods. Decentralized public health infrastructure has historically given local governments in the Philippines discretion in which services are offered, resulting in local FP limitations [19]. In theory, the 2012 legislation resolved this by actively requiring the provision of a full range of contraceptive methods; however, as that legislation only took full effect in 2017, decentralization continues to be cited as a barrier to women having consistent access to midwives with comprehensive FP training [11,18,20,21]. No national review of midwives’ FP training and skills has been conducted [22]. The need for further training was reflected in this study, as numerous participants referenced gaps in their own or colleagues’ FP training.

The potential impact of provider bias within the Philippines’ cultural and religious context is an important consideration. Literature to date has been mixed regarding the impact of such bias. A 2016 study interviewed midwives regarding their moral and political perspectives on FP; they described promoting FP and educating patients on FP options [23]. Another study hypothesized that provider perceptions may limit counseling provision, citing a 32% rate of missed FP counseling opportunities at contraception clinics [18]. Biases among midwives that would pose a barrier to patient FP education and access were not identified in this study. Based on this study and other literature, training gaps play a much larger role in FP trends than possible provider biases.

Social and cultural factors feature prominently in the literature as ongoing challenges. Social norms and concerns about contraceptive methods pose persistent barriers [8,11,18]. In this study, the role of influential community members in reinforcing misconceptions was emphasized, as midwives described patients frequently citing FP superstitions. These are not barriers easily addressed by training, supply chain improvement, or other systematic changes. However, the midwives in our study described their strategies for addressing these misconceptions at an individual level and reinforcing this teaching over time.

Banas have been consistently found to play a significant role in FP decisions and utilization. One study assessing the autonomy of women found that partners’ approval of contraception was associated with a lower risk of mistimed births, reinforcing the importance of a mutual decision [14]. This was affirmed by the midwives in this study, who repeatedly spoke about the importance of counseling both partners and ensuring that the bana was agreeable to his partner’s preferred method.

The role of religion remains a complex challenge. Recent studies have consistently found that Filipinas do not cite religion as a reason for not using modern contraception [8,12,18]. This study’s findings were consistent in that the Catholic or Christian religion was not cited by any midwife as affecting patients’ FP decisions, nor was Islam, although the midwives did reflect different considerations between the two religions. While individual religious beliefs have consistently been found not to be a barrier to contraception, Catholicism remains the predominant religion in the Philippines, which has continued implications for public education and policy changes.

Strengths and limitations

This study centered on the perspective of midwives, who serve as the primary women’s health practitioners throughout much of the Philippines yet have not previously been the focus of a qualitative study that explores FP access and utilization. The diversity of clinic structure and geographic setting permitted a range of care settings to be reflected in the interviews. Flexibility inherent within the semi-structured interview methodology allowed for conversations to focus on aspects considered most important by each midwife. The observational time spent by the primary investigator provided a meaningful context for the Filipino maternal healthcare delivery system and served to validate the main findings and themes.

The study population was derived from three clinics within one region, which may limit the generalizability of the findings. However, diversity in both clinic and practice settings was pursued, and the major findings did reach a point of thematic saturation, in keeping with a standard qualitative methodology. All participants were assured of the anonymity of their interview content, but there could have been a reluctance to share certain information. Many of the encounters observed for triangulation did not occur in English, limiting some observational data to English words interspersed in these encounters and interpretation by midwives following the encounters, introducing the possibility for encounter content to be reflected less objectively.

## Conclusions

This study gives voice to a previously missing perspective on modern contraceptive use, that of the midwives at Filipino birthing clinics. It illustrates the importance of the Filipino public health infrastructure, anchored in the BHS, operating consistently and in a standardized fashion. It highlights the significance of cultural and social factors in FP decisions and the importance of midwives providing education and guidance within that context. It emphasizes the necessity of continued and enhanced education, beginning in schools, regarding the fertility cycle and benefits of FP. It reinforces that religion does not change an individual woman’s FP preferences or decisions. Finally, it depicts a lack of provider bias negatively impacting patient access to modern contraceptive methods. Each of these aspects parallels and supports existing literature from qualitative studies of Filipinas and their FP perspectives. Building on these findings, further exploration of the inventory tracking and supply delivery systems in place for the BHS network would be valuable in assessing opportunities for improved method availability. Additionally, further study of current school- and community-based educational initiatives, including content and educational outcomes, would help guide future work aiming to address educational needs regarding fertility and FP.
